# Epidemic of illicit drug use, mechanisms of action/addiction and stroke as a health hazard

**DOI:** 10.1002/brb3.7

**Published:** 2011-09

**Authors:** Katherine Esse, Marco Fossati-Bellani, Angela Traylor, Sheryl Martin-Schild

**Affiliations:** 1 Stroke Program, Department of Neurology, Tulane University School of Medicine1440 Canal Street, TB-52, Suite 1000, New Orleans, Louisiana 70112-2715; 2 Department of Internal Medicine, Tulane University School of Medicine1440 Canal Street, TB-52, Suite 1000, New Orleans, Louisiana 70112-2715; 3 Department of Psychiatry & Behavioral Sciences, Tulane University School of Medicine1440 Canal Street, TB-52, Suite 1000, New Orleans, Louisiana 70112-2715

**Keywords:** Acute ischemic stroke, illicit drugs, intracerebral hemorrhage, subarachnoid hemorrhage, substance abuse

## Abstract

Drug abuse robs individuals of their jobs, their families, and their free will as they succumb to addiction; but may cost even more: a life of disability or even life lost due to stroke. Many illicit drugs have been linked to major cardiovascular events and other comorbidities, including cocaine, amphetamines, ecstasy, heroin, phencyclidine, lysergic acid diethylamide, and marijuana. This review focuses on available epidemiological data, mechanisms of action, particularly those leading to cerebrovascular events, and it is based on papers published in English in PubMed during 1950 through February 2011. Each drug's unique interactions with the brain and vasculature predispose even young, healthy people to ischemic or hemorrhagic stroke. Cocaine and amphetamines have the strongest association with stroke. However, the level of evidence firmly linking other drugs to stroke pathogenesis is weak. Large epidemiological studies and systematic evaluation of each drug's action on the brain and cardiovascular system are needed to reveal the full impact of drug use on the population.

## Introduction

Approximately 2.2 million people reported current illicit drug use in the 2009 United States National Health Survey (Substance Abuse and Mental Health Services Administration 2010). Almost one million emergency department (ED) visits in the United States involved illicit drugs in 2007 (Drug Abuse Warning Network 2010). Though it is difficult to clearly associate deaths directly to drug exposure, an estimated 17,000 deaths were attributed to illicit drug use in 2000 (Mokdad et al. 2004). The major causes of death from drugs are overdose, suicide, AIDS, and accidents; however, cerebrovascular disease is a significant source of morbidity from drug use. Drug use may be the most common predisposing condition for stroke among patients under 35 years of age. In fact, drug abusers aged 15 to 44 years were 6.5 times more likely to have a stroke than nondrug users (Kaku and Lowenstein 1990). Of 422 patients aged 15–44 with acute ischemic stroke (AIS), 12.1% were found to have recent drug use and 4.7% had drug abuse as the likely cause of stroke (Sloan et al. 1998). Lastly, a large population-based study of 1935 patients with stroke diagnoses revealed that 14.4% of intracerebral hemorrhages (ICH) and 14.4% of AISs were associated with drug use (Westover et al. 2007). Despite this obvious connection between illicit drugs and stroke, epidemiologic data are hard to find. Most evidence that illegal drugs are risk factors for stroke is anecdotal (Brust 2002). Using the data from a number of case studies and a limited number of population studies, this article will outline various illicit drugs and their association to AIS, ICH, and subarachnoid hemorrhage (SAH).

The main illicit drugs associated with stroke are cocaine, amphetamines, Ecstasy, heroin/opiates, phencyclidine (PCP), lysergic acid diethylamide (LSD), and cannabis/marijuana. Tobacco and ethanol are also associated with stroke, but will not be discussed here. This article will outline current epidemiology, pharmacology, evidence related to strokes, and mechanisms of action related to stroke risk for each drug listed above. The table summarizes proposed stroke mechanisms for each reviewed drug and stroke subtype.

## Search strategy and selection criteria

References for this review were identified by searches of PubMed from 1950 until February 2011 with the terms “ischemic stroke,”“intracerebral hemorrhage,”“subarachnoid hemorrhage,”“illicit drugs,”“substance abuse,”“cocaine,”“amphetamines,”“heroin,”“marijuana,”“phencyclidine,”“lysergic acid diethylamide,” and “Ecstasy.” Articles were also identified through searches of the authors’ own manuscripts and relevant publications. Only papers published in English were reviewed.

## Associated Drugs

### Cocaine

In the 1970s, recreational use of cocaine became widespread due to the production of crack cocaine, a purer and cheaper form of cocaine. The late 1980s saw an epidemic of cocaine: 30 million people of all socioeconomic backgrounds were cocaine users and 6 million were cocaine addicts (Agarwal and Sen 2010). In 2009, cocaine was the second-most commonly used illicit drug in the United States after marijuana. Of one million illicit drug-related ED visits yearly in the United States, nearly half are related to cocaine, making cocaine the most frequent cause of illicit drug-related ED visits (The DAWN report 2010).

#### Pharmacology

Cocaine comes in two chemical forms: the hydrochloride salt, which is the powdered form of cocaine that is water soluble, and cocaine alkaloid, a free base that is lipid soluble. The effects of cocaine include local anesthesia, vasoconstriction, and central nervous system stimulation. Cocaine prevents neurotransmitter (dopamine, norepinephrine, serotonin, and acetylcholine) reuptake at presynaptic nerve terminals, thereby increasing the amounts of neurotransmitters available for stimulation of sympathetic nerves. The euphoria related to cocaine use is a result of accumulation of dopamine and serotonin in the mesolimbic and mesocortical areas of the brain (Treadwell and Robinson 2007). These reward circuits are related to drug-seeking behavior, addiction, and dependence, making cocaine one of the most potent and highly addictive chemicals (Goforth et al. 2010).

**Table:** 

Stroke Mechanisms by Drug and Stroke Type			

	Acute Ischemic Stroke	Intracerebral Hemorrhage	Subarachnoid Hemorrhage
Cocaine	• Vasospasm	• Hypertensive surges	• Aneurysm rupture from hypertensive surges
	• Enhanced platelet aggregation	• Mycotic aneurysm rupture from cardioembolic event	• Facilitation of aneurysm formation
	• Vasculitis	• Hemorrhagic transformation of embolism	
	• Accelerated atherosclerosis		
	• Cardioembolism		
	○ Arrhythmia		
	○ Cardiomyopathy		
	○ Septic embolism		
Amphetamines	• Cardioembolism	• Hypertensive surge	• Aneurysms
	○ Arrhythmias with thrombosis		
	○ Cardiomyopathy		
	• Vasculitis		
	• Microinfarcts from blood vessel injury and accelerated atherosclerosis		
Ecstasy	• Cardioembolism from:	• Hypertensive surge	• Aneurysm formation and rupture
	○ Arrhythmias	• Vasodilation from decreased serotonin from chronic use	
	○ Cardiomyopathy	• Consumptive coagulopathy = spontaneous bleed	
	• Vasospasm → Vessel wall damage		
	• Direct vasoconstrictive effect of Serotonin		
	• Hyperthermia→Clotting cascade/DIC → microinfarcts		
Heroin/Opiates	• Cardioembolism·	None	None
	○ Endocarditis		
	○ Foreign bodies		
	• Arteritis/Vasculitis		
	• Hypotension/Hypoxemia		
PCP	• Vasospasm? (only in vitro)	• Hypertensive effect	• Weakened arterial walls
LSD	• Vasospasm	None	None
	• Direct vasoconstrictive effect of Serotonin		
Marijuana	• Hypotension?	None	None
	• Vasospasm?		
	• Cardioembolism from arrhythmias?		

#### Cocaine and stroke

As crack cocaine use increased in the 1970s and 1980s, case reports of cocaine-associated AIS, ICH, and SAH became more prevalent, which is especially striking given the young age at which strokes occurred. A review article in the late 1980s found that the mean age of patients with cocaine-related stroke was 32.5 years (Klonoff et al. 1989).

Case series characterizing the brain location and etiology of each type of cocaine-related stroke have been performed. Cocaine-associated AISs have been reported in nearly every vascular territory in the brain; anterior circulation, posterior circulation, spinal cord, brainstem, and retina have been affected (Brust 2002). Both cortical and subcortical strokes can occur (Daras et al.; Jacobs et al. 1989). The etiology of ischemic infarcts varies as well; large artery, small artery, and cardioembolic strokes all appear to be of relatively equal incidence (Martin-Schild et al. 2009).

While AIS is far more common than ICH or SAH overall, the frequency of hemorrhagic stroke is disproportionately high in cocaine-related strokes (Treadwell and Robinson 2007). Intracerebral hemorrhages are found throughout the brain, including basal ganglia, thalamus, lobar, brainstem, and cerebellar locations. While one study found mostly lobar locations (73% of 34 patients) (Kaku and Lowenstein 1990), a recent study of 45 cocaine users with ICH found predominantly ICH in the basal ganglia (Martin-Schild et al. 2010). This may depend on the prevalence of underlying hypertension in different populations. The prevalence of underlying vascular lesions in patients with cocaine-related ICH has been variable, ranging from 10% (Martin-Schild et al. 2010) to nearly 50% related to ruptured aneurysms or arterio-venous malformations (AVMs) (Brust 2002; Enevoldson 2004).

#### Mechanisms of strokes

The main etiologies that have been suggested include hypertensive surges, vasospasm, enhanced platelet aggregation, cerebral vasculitis, accelerated atherosclerosis, and cardioembolism (Treadwell and Robinson 2007).

Chronic uncontrolled hypertension is a major risk factor for stroke. Repeated use of cocaine can raise blood pressure, increasing the risk for stroke, even in patients who do not have baseline hypertension. Hypertensive surges may be responsible for the majority of hemorrhagic strokes associated with cocaine use.

Vasospasm is a fascinating mechanism for cocaine-induced stroke. Defined as sudden and usually reversible changes in vascular caliber due to vascular smooth muscle changes, vasospasm is more commonly encountered as a complication of SAH. A case study of cocaine users, however, found tunica media and elastic lamina damage in vessels in multiple locations in the brain possibly due to chronic vasospasm (Konzen et al. 1995). Radiographic studies (Kaufman et al. 1998) confirmed animal studies (He et al. 1994) that demonstrated a dose-dependent vasoconstriction of cerebral vessels on magnetic resonance angiography in response to cocaine.

The pathophysiology of vasospasm in cocaine use is multifactorial. Cocaine has effects on the calcium channels in smooth muscle cells in vascular walls. It promotes the release of calcium from the sarcoplasmic reticulum and also may allow influx of external calcium into the smooth muscle cells, causing contraction of the vessel walls. Accumulation of catecholamines caused by prevention of reuptake may result in smooth muscle contraction by an effect on several receptors in the vessel walls. This latter mechanism is supported by dopamine antagonist (haloperidol) and calcium channel blocker (verapamil) prevention of vasospasm in cocaine-exposed smooth muscle cells (He et al. 1994). Endothelin-1, an endogenous vasoactive peptide, has been implicated in the development of atherosclerosis and vasoconstriction. Endothelin-1 has been detected in the urine and serum of cocaine users. An endothelin-1 antagonist reversed cocaine-induced vasospasm in animal models (Fandino et al. 2003). Vasoconstriction may play a role in cocaine-related stroke even days after last cocaine use. Metabolized by the liver, cocaine has a half-life of approximately 1 hour, but major provasoconstrictive metabolites can last for days. There is also large variation among individuals, with metabolites lingering in chronic users for up to 3 weeks (Enevoldson 2004).

Vasospasm may cause endothelial injury, resulting in intimal hyperplasia and platelet activation and aggregation, ultimately occluding vessels (Treadwell and Robinson 2007). This may be why microvascular white matter changes are found on MRI in chronic cocaine users (Volkow et al. 1988; Goforth et al. 2010). Cocaine administration activates platelets resulting in α-granule release and the formation of platelet-rich microaggregates (Heesch et al. 2000). It also increases platelet responsiveness to arachidonic acid, and causes the release of thromboxane β2 and plasminogen activating factor-1 inhibitor (PAI-1). All of these factors promote platelet aggregation (Togna et al. 1985; Kolodgie et al. 1995) and facilitation of thrombus formation.

Very few cases of biopsy-proven vasculitis associated with cocaine exposure have been reported. These cases describe a hypersensitivity-type vasculitic morphology that differs from the typical inflammatory central nervous system vasculitis. Supporting this is the fact that in cases of presumed cocaine-induced vasculitis, steroids failed to improve the patient's symptoms in the short term (Merkel et al. 1995). Most studies have failed to demonstrate these findings on autopsy (Brust 2002; Enevoldson 2004).

Cocaine may promote accelerated atherosclerosis, leading to longer term increased risk for AIS in cocaine users. Rabbits with high cholesterol that were exposed to cocaine demonstrated a greater extent of cholesterol plaque in the proximal thoracic aorta than control rabbits (Kolodgie et al. 1993). In the presence of cocaine, cell membranes are more permeable to atherogenic lipoproteins (Kolodgie et al. 1993, 1995, 1999).

Cardioembolism is a well-known cause of cocaine-related stroke. Mechanisms of embolism and ischemic stroke include infective endocarditis in patients who inject cocaine hydrochloride, arrhythmia, acute and chronic dilated cardiomyopathy, and myocardial infarction (Sloan and Mattioni 1992). Cocaine use is highly associated with infective endocarditis: in one study of drug users with endocarditis, 79% were cocaine users (Chambers et al. 1987). In addition to embolization, endocarditis can cause a septic cerebral arteritis. Endocarditis provokes ICH from rupture of mycotic aneurysms and hemorrhagic transformation of embolic stroke (Hart et al. 1987; Enevoldson 2004; Hagan and Burney 2007).

Hypertensive surge with or without an underlying vascular malformation is the most common implicated etiology for ICH and SAH. The indirect sympathomimetic effects of cocaine transiently raise the systolic blood pressure, which can cause spontaneous bleeding in existing AVMs, aneurysms, or areas of old ischemic strokes, or may actually facilitate aneurysm formation (Nolte et al. 1995). Cocaine users with ICH have very high blood pressure on admission (Martin-Schild et al. 2009), and have blood in classic hypertensive locations. Brainstem hemorrhages were over-represented in patients with cocaine-associated ICH. Cocaine users with ICH have worse short-term functional outcome compared to patients with hemorrhage who are not cocaine users. In fact, cocaine users with ICH were nearly five times more likely to be dependent and three times more likely to die than patients with ICH who did not use cocaine (Martin-Schild et al. 2010). When SAH occurs in cocaine users, aneurysms are often detected on angiography (Oyesiku et al. 1993; Fessler et al. 1997).

### Amphetamines

Widespread amphetamine abuse began during World War II, when it was offered to soldiers to fight fatigue and improve morale. By the 1950s, there was an upswing in legal prescription of amphetamines in the United States. The manufacture and distribution of amphetamines was greatly reduced after the passage of the Controlled Substances Act in 1970. In the late 1980s and 1990s, however, amphetamines were back in vogue, due to the ease and low expense of synthesizing methamphetamines in amateur laboratories. As of 2000, an estimated 35 million people abused amphetamines worldwide, as compared with 15 million cocaine abusers (Albertson et al. 2007).

#### Pharmacology

Amphetamines constitute a group of drugs with chemical similarity to the natural neurotransmitters epinephrine and dopamine. Synthetic modifications result in differing effects and properties. Each is a weak base and can be absorbed via multiple routes. Depending on the particular drug and dosage, the half-life can range from 10 to 30 hours. Methamphetamine (meth) is the most potent of amphetamines and is most commonly abused; it has a half-life of 12 hours, is metabolized through the liver, and has an active metabolite which is a potent hallucinogen.

Amphetamines block the presynaptic reuptake of the catecholamines dopamine, norepinephrine, and serotonin, allowing these neurotransmitters to remain in the synapse to trigger and saturate the postsynaptic receptors. The user feels euphoric and experiences increased motor movements, increased productivity, decreased appetite, and increased libido (Albertson et al. 1999; Winslow et al. 2007; Freye and Levy 2009). Amphetamines create strong effects of addiction, craving, and tolerance in chronic users (Freye and Levy 2009). Amphetamines have wide-ranging effects on nearly every organ system. Amphetamines have been linked to myocardial infarction, cardiomyopathy, renal failure, liver failure, respiratory failure, stroke, memory loss, confusion, and a many psychiatric symptoms.

#### Amphetamines and stroke

Amphetamine use increases the odds of stroke by almost four times that of nonusers (Petitti et al. 1998) and results in greater disability and mortality rates (Westover et al. 2007). AIS, ICH, and SAHs have been reported in the literature. Most case series report a disproportionate rate of hemorrhagic stroke with amphetamine use, up to twice the risk of cocaine (odds ratio 4.95 vs. 2.33) (Westover et al. 2007).

#### Mechanisms of stroke

Amphetamines, like cocaine, are sympathomimetic. Therefore, the mechanisms of stroke in amphetamine users are similar to those of cocaine-related strokes. Up to 75% of patients with methamphetamine-related stroke have significantly elevated blood pressures on arrival (Perez et al. 1999). Amphetamines may accelerate hypertensive heart disease with myocardial hypertrophy and interstitial fibrosis and cause direct myocardial toxicity with contraction-band necrosis (Yeo et al. 2007; Yi et al. 2008; Ito et al. 2009). Cardiomyopathy is a well-established complication of amphetamine abuse. Methamphetamine use is associated with a 3.7-fold increase in the odds of detecting cardiomyopathy (95% confidence interval: 1.8–7.8) (Yeo et al. 2007). Methamphetamine users with cardiomyopathy have lower left ventricular ejection fractions and higher end-diastolic and left atrial volumes than heart failure patients without methamphetamine use (Ito et al. 2009). Cardiomyopathy results in arrhythmias and thrombosis, leading directly to cardio-embolic strokes.

Unlike cocaine, an association between chronic amphetamine use, stroke and vasculitis have been reported. Angiography in multidrug abusers detected findings consistent with necrotizing periarteritis in multiple organs on angiography, and confirmed those findings, specifically in the central nervous system on autopsy of select cases. Amphetamines were the most commonly abused drug in these studies (Citron et al. 1970; Halpern and Citron 1971; Margolis and Newton 1971; Stafford et al. 1975; Wooten et al. 1983; Salanova and Taubner 1984; Shibata et al. 1991; Brust 1997; Ho et al. 2009).

Acute increase in systolic blood pressure during amphetamine use leads to spontaneous ICH (McGee et al. 2004). In fact, hemorrhagic strokes have been observed after only a single use of amphetamines (Stoessl et al. 1985). Most cases of ICH involve brain regions commonly affected in hypertensive ICH (Ho et al. 2009). Histopathological evidence support that repeated amphetamine abuse can result in blood vessel injury, leading to vessel wall necrosis, microinfarcts in small vessels, and atherosclerosis (McGee et al. 2004; Ho et al. 2009). Amphetamine-related SAHs mostly frequently report underlying aneurysms (Ho et al. 2009; Kaku and Lowenstein 1990).

### Ecstasy

Ecstasy is a nonspecific name for a variety of “designer drugs” used mainly by young adults. They are derivatives of amphetamine. The majority of Ecstasy in use is 3,4-methylenedioxymeth-amphetamine, or MDMA, N-ethyl-3,4-methylenedioxyamphetamine or MDEA (sometimes called “Eve”), or 3.4-methylenedioxyamphetamine (MDA). Recent epidemiological data indicate that Ecstasy use is increasing among college students (Strote et al. 2002).

#### Pathophysiology

Though Ecstasy is derivative of amphetamine, the drug more closely resembles the hallucinogen mescaline, rendering it to be a blend of hallucinogenic and catecholaminergic effects. Ecstasy increases the release of, and inhibits the reuptake of, serotonin and norephinephrine, with lesser effects on dopamine. The toxicity, time course, and intensity of reaction can differ significantly between preparations. The drug is usually ingested in pill form, metabolized by the liver, and generally achieves peak concentration in the blood approximately two hours after ingestion.

#### Ecstasy and stroke

There are no epidemiological studies specifically studying the incidence of Ecstasy-related strokes. There are a small number of case studies of both ischemic and hemorrhagic strokes occurring within hours of ingestion of Ecstasy (Hughes et al. 1993; Harries and De Silva 1992; Gledhill et al. 1993; Manchanda and Connolly 1993; Hanyu et al. 1995; Kalant 2001; Auer et al. 2002).

#### Mechanisms of stroke

The vascular distribution of AISs related to Ecstasy use is variable. The possible etiologies of MDMA-induced stroke are similar to those of cocaine- and amphetamine-related strokes. Cardiac arrhythmias, have been implicated in Ecstasy-related cardiac death, and are potential causes of Ecstasy-related stroke via cardioembolism (Hughes et al. 1993). Cardiomyopathy has also been described in Ecstasy users and is associated with congestive heart failure, arrhythmia, and stroke.

After exposure to Ecstasy, vasospasm and necrosis have been observed in the vasculature of the globus pallidus and occipital cortex, making damage from AIS most likely in these areas (Reneman et al. 2000; Rojas et al. 2005; Hagan and Burney 2007). Damage to the vessel walls over time from chronic vasospasm and necrosis may also lead to both thrombosis and aneurysmal dilatation of cerebral vessels. This can then lead to AIS, ICH, or SAH (Kalant 2001; Auer et al. 2002). Ecstasy increases the bioavailability of serotonin, a potent vasoconstrictive amine, which decreases cerebral blood flow and can lead to cerebral infarction (Hanyu et al. 1995). Long-term use of Ecstasy results in decreased overall serotonin availability and vasodilation, and even ICH in the setting of hypertension (Reneman et al. 2000).

High fever, provoked by Ecstasy's activation of the hypothalamus, may trigger the clotting cascade, resulting in disseminated intravascular coagulation and microinfarcts throughout the body, including the brain, as well as bleeding due to consumptive coagulopathy (Kalant 2001; Freye and Levy 2009). Very little evidence supports vasculitis as a complication of Ecstasy use (Manchanda and Connolly 1993).

Hypertensive surge may lead to small-vessel ICH or large-vessel hemorrhage via rupture of an underlying cerebrovascular malformation. Esctasy-related ICH occurs in regions commonly affected by hypertension, and SAH is usually associated with an underlying aneurysm.

### Opiates/Heroin

Heroin is a semi-synthetic derivative of opium. Heroin addiction became a problem around the turn of the 20th century. The United States Department of Health and Human Services’ National Household Survey on Drug Abuse Study estimated that in 2008, 3.8 million people over the age of 12 had used heroin during their lifetime. In 2009, 180,000 people in the United States used heroin for the first time, representing a significant increase from prior years (Substance Abuse and Mental Health Services Administration 2010).

#### Pharmacology

Heroin binds to endogenous opiate receptors (mu, kappa, and delta) located throughout the body, including the brain and the spinal cord. The mu receptor is responsible for analgesia, euphoria, nervous system depression, respiratory depression, and constipation. Heroin, unlike morphine, is able to cross the blood–brain barrier very easily. Heroin tends to cause hypotension from decreased peripheral vascular resistance, bradycardia by inhibiting the baroreceptor reflex, and respiratory depression by slowing the brain's response to high CO_2_ and low oxygen levels. When heroin is injected, the initial effect, or “rush,” occurs within a few minutes and peaks at around 10 minutes. After this, sedation ensues and lasts about one hour.

#### Stroke and heroin

Heroin and other opiates are known to cause severe morbidity and death from violence, overdose, AIDS, suicide, and sepsis. However, strokes associated with heroin/opiate use are rarely reported. Despite this scarce reporting, opiates were 16 times less likely to cause hemorrhagic strokes and five times less likely to cause ischemic stroke than amphetamines (Westover et al. 2007). Most reported strokes associated with heroin use are ischemic (Hagan and Burney 2007).

#### Mechanisms of stroke

Heroin-associated stroke is most often due to cardioembolism in the setting of infective endocarditis (Hagan and Burney 2007). Another source for embolic disease from heroin use is foreign bodies that have been added to the heroin. Foreign bodies (potentially including starch, sugar, Ajax, quinine, lactose, mannitol, caffeine, aspirin, lidocaine, strychnine, and talcum powder) enter the circulation and become lodged in the lungs. A granulomatous reaction leads to pulmonary hypertension, causing or exacerbating right-to-left pulmonary shunts predisposing to cardioembolic strokes (Brust 1989; Lucas 2005).

Arteritis and vasculitis have also been indirectly implicated as a cause of heroin-related strokes. “Beading” on angiography along with supporting laboratory studies has been reported, but pathological evidence supporting this theory is lacking (Brust 1997) and it is not known if the vessel changes are in response to the heroin itself or adulterants. Other potential causes of stroke include hypotension and hypoxemia induced by opiate overdose; these can result in global hypoxic-ischemic injury to classically vulnerable areas of the brain (Andersen and Skullerud 1999).

### Phencyclidine (PCP)

Phencyclidine, known as PCP, is classified as a dissociative anesthetic similar to ketamine. The drug was initially introduced as an anesthetic that did not paralyze the diaphragm or cause respiratory depression, but it was pulled from the market due to reports of adverse neuropsychiatric reactions after anesthesia. PCP use has declined over time (Lerner and Burns 1978; Gahlinger 2004). The lifetime prevalence of PCP use in the United States was estimated at approximately 6.6 million people over the age of 12 (Substance Abuse and Mental Health Services Administration 2010).

#### Pharmacology

The full range of PCP effects on the brain are incompletely understood, due to effects on multiple neurotransmitter pathways and receptors including N-methyl D-aspartate (NMDA) (antagonist) nicotinic acetylcholine (antagonists) and dopamine (agonist). Complicating the picture further, PCP may have its *own* receptors on cerebral vessels (Altura et al. 1983). Since PCP is stored in the body's fat, re-mobilization from those stores can cause recurrent symptoms for days to months. PCP is metabolized by the liver, and has multiple metabolites. Ten percent of the dose of phencyclidine is excreted unchanged in the urine, and can be picked up by a urine drug screen (Domino 1978; Gahlinger 2004; West et al. 2011).

#### PCP and Stroke

A total of five cases of PCP-associated stroke were found in the literature—all of them were hemorrhagic. PCP's sympathomimetic hypertensive effect may be the provoking factor. Hypertension is one of the primary clinical findings in PCP intoxication (McCarron et al. 1981). Spikes of severe hypertension can occur hours to days after use. PCP-related SAH has been reported and may result from weakening of arterial walls (Boyko et al. 1987). While vasospasm can be provoked in vitro by PCP (Altura et al. 1983) in a dose-dependent manner at concentrations paralleling those of patients who overdosed on the drug, there are no reported cases confirming vasospasm in association with stroke in PCP users.

### LSD

Lysergic acid diethylamide, or LSD, is a potent hallucinogen. During the 1950s to 1970s, the psychiatric community extensively explored the use of LSD to treat psychosis, alcohol addiction, autism, and criminal behavior. Its current popularity is declining: the number of new users dropped from 958,000 in 2000 to 337,000 in 2009 (Mechem and Hall 2008; Substance Abuse and Mental Health Services Administration 2010).

#### LSD and Stroke

Only four cases of stroke related to LSD have been reported in the literature. All of the cases involved AIS in patients under the age of 25 (Sobel et al. 1971; Lieberman et al. 1974). The two cases in which LSD was the sole drug used by the patients were cases that involved large-artery occlusions. Similar to ergot alkaloids, LSD affects serotonin receptors and may cause vessel constriction. In vitro, LSD produces significant vasospasm of cerebral arteries; this effect is reversed by a 5-HT antagonist (methysergide) or a calcium channel blocker (verapamil) (Altura and Altura 1981). Given the apparent ability of LSD to cause vasospasm in vitro, it is more likely that a vasospastic process is responsible for LSD-related strokes (Altura and Altura 1981).

### Marijuana

Marijuana is the most commonly used recreational drug in the United States, and 15 states have approved marijuana for medical use (State Medical Marijuana Laws 2010). More than 16.7 million people reported marijuana use within the past month on a national survey conducted in 2009 (Substance Abuse and Mental Health Services Administration 2010).

#### Marijuana and Stroke

Evidence supporting marijuana's role in stroke is scarce, considering its widespread use. One study demonstrated an odds ratio for AIS with marijuana use of 1.76 (95% confidence interval 1.15–2.71), even when controlling for other risk factors (Kaku and Lowenstein 1990). Twenty-one cases of imaging-positive stroke related to marijuana use have been reported (Cooles and Michaud 1987; Zachariah 1991; Barnes et al. 1992; Lawson and Rees 1996; McCarron and Thomas 1997; Mouzak et al. 2000; Mesec et al. 2001; Mathew et al. 2003; Finsterer et al. 2004; Geller et al. 2004; Moussouttas 2004; Mateo et al. 2005; Aryana and Williams 2007; Duchene et al. 2010; Renard et al. 2010). Twenty were ischemic infarcts in men; one was an ischemic infarct in a woman (Duchene et al. 2010). No consistent pattern of infarct distribution was identified.

Proposed mechanisms for marijuana-associated cerebral infarction include hypotension, vasospasm, and arrhythmia with resulting cardioembolism (Cooles and Michaud 1987; Mathew et al. 2003; Geller et al. 2004; Moussouttas 2004; Mateo et al. 2005; Aryana and Williams 2007). Since these phenomena are often transient, the direct role in stroke is elusive. Cannabinoids have a role in cerebral autoregulation, vascular tone, and cardiac pathology (Mittleman et al. 2001; Mathew et al. 2003; Moussouttas 2004) and may provoke the reversible vasoconstriction syndrome associated with thunderclap headache, SAH, ICH, and cerebral ischemia (Ducros et al. 2007).

## Conclusion

Illicit drugs are used by millions of people every day. Based on an extensive review of the medical literature, it is apparent that these illicit drugs are dangerous for many reasons, and some of them appear to increase a person's risk for both ischemic and hemorrhagic strokes. The evidence is fairly clear that cocaine and amphetamines are strongly linked to stroke, but Ecstasy, opiates, phencyclidine, LSD, and marijuana simply do not have the burden of evidence required to firmly link usage to stroke pathogenesis. Unfortunately, the lack of standardization and the propensity for many of these drugs to be mixed with adulterants has muddied the picture of how these drugs act in the body. Further, the study of illicit drugs is hampered by the need for patient or surrogate disclosure or reliance on urine toxicology for which commonly used medications may result in a falsely positive urine drug test (Brahm et al. 2010). Regardless, future studies are needed to systematically evaluate how each of these chemicals acts on the cerebrovascular system. In addition, the lack of epidemiological studies on drugs and stroke hinders the ability of researchers to gain perspective on the impact that drug use may have on the population. Going forward, research on illicit drugs and their relationship to stroke and other morbidities is a responsibility that cannot be denied by those devoted to reducing the burden of stroke and cardiovascular health on society.

## References

[b1] Agarwal P, Sen S (2010). Neurologic effects of cocaine: eMedicine neurology. [homepage on the Internet]. http://emedicine.medscape.com/article/1174408-overview.

[b2] Albertson TE, Derlet RW, Van Hoozen BE (1999). Methamphetamine and the expanding complications of amphetamines. West J. Med..

[b3] Albertson TE, Kenyon NJ, Morrissey B, Shannon M, Borron SW, Burns M (2007). Amphetamines and Derivatives. Haddad and Winchester's clinical management of poisoning and drug overdose [Internet].

[b4] Altura BT, Altura BM (1981). Phencyclidine, lysergic acid diethylamide, and mescaline: cerebral artery spasms and hallucinogenic activity. Science.

[b5] Altura BT, Quirion R, Pert CB, Altura BM (1983). Phencyclidine (“angel dust”) analogs and sigma opiate benzomorphans cause cerebral arterial spasm. Proc. Natl. Acad. Sci. USA.

[b6] Andersen SN, Skullerud K (1999). Hypoxic/ischaemic brain damage, especially pallidal lesions, in heroin addicts. Forensic Sci. Int..

[b7] Aryana A, Williams MA (2007). Marijuana as a trigger of cardiovascular events: speculation or scientific certainty?. Int. J. Cardiol..

[b8] Auer J, Berent R, Weber T, Lassnig E, Eber B (2002). Subarachnoid haemorrhage with “ecstasy” abuse in a young adult. Neurol. Sci..

[b9] Barnes D, Palace J, O'Brien MD (1992). Stroke following marijuana smoking. Stroke.

[b10] Boyko OB, Berger PC, Heinz ER (1987). Pathological and radiological correlation of subarachnoid hemorrhage in phencyclidine abuse. Case report. J. Neurosurg..

[b11] Brahm NC, Yeager LL, Fox MD, Farmer KC, Palmer TA (2010). Commonly prescribed medications and potential false-positive urine drug screens. Am. J. Health Syst. Pharm..

[b12] Brust JC (2002). Neurologic complications of substance abuse. J. Acquir. Immune Defic. Syndr..

[b13] Brust JC (1997). Vasculitis owing to substance abuse. Neurol. Clin..

[b14] Brust JCM, Vinken PJ, Bruyn GW, Klawans HL, Toole JF (1989). Stroke and drugs. Handbook of clinical neurology: Vascular diseases, part III.

[b15] Chambers HF, Morris DL, Täuber MG, Modin G (1987). Cocaine use and the risk for endocarditis in intravenous drug users. Ann. Intern. Med..

[b16] Citron BP, Halpern M, McCarron M, Lundberg GD, McCormick R, Pincus IJ (1970). Necrotizing angiitis associated with drug abuse. N. Engl. J. Med..

[b17] Cooles P, Michaud R (1987). Stroke after heavy cannabis smoking. Postgrad. Med. J..

[b18] Daras M, Tuchman AJ, Marks S Central nervous system infarction related to cocaine abuse. Stroke.

[b19] Domino EF (1978). Neurobiology of phencyclidine—an update. NIDA Res. Monogr..

[b20] Drug Abuse Warning Network (2010). DAWN emergency department publications: 2007: National estimates of drug-related emergency department visits. http://dawninfo.samhsa.gov/pubs/edpubs/.

[b21] Duchene C, Olindo S, Chausson N, Jeannin S, Cohen-Tenoudji P, Smadja D (2010). Cannabis-induced cerebral and myocardial infarction in a young woman. Rev. Neurol. (Paris).

[b22] Ducros A, Boukobza M, Porcher R, Sarov M, Valade D, Bousser M-G (2007). The clinical and radiological spectrum of reversible vasoconstriction syndrome. A prospective series of 67 patients. Brain.

[b23] Enevoldson TP (2004). Recreational drugs and their neurologic consequences. J. Neurol. Neurosurg. Psychiatry.

[b24] Fandino J, Sherman JD, Zuccarello M, Rapoport RM (2003). Cocaine-induced endothelin-1-dependent spasm in rabbit basilar artery in vivo. J. Cardiovasc. Pharmacol..

[b25] Fessler RD, Esshaki CM, Stankewitz RC, Johnson RR, Diaz FG (1997). The neurovascular complications of cocaine. Surg. Neurol..

[b26] Finsterer J, Christian P, Wolfgang K (2004). Occipital stroke shortly after cannabis consumption. Clin. Neurol. Neurosurg..

[b27] Freye E, Levy JV (2009). Pharmacology and abuse of cocaine, amphetamines, ecstasy and related designer drugs: A comprehensive review on their mode of action, treatment of abuse and intoxication [Internet].

[b28] Gahlinger PM (2004). Club drugs: MDMA, gamma-hydroxybutyrate (GHB), Rohypnol, and ketamine. Am. Fam. Physician.

[b29] Geller T, Loftis L, Brink DS (2004). Cerebellar infarction in adolescent males associated with acute marijuana use. Pediatrics.

[b30] Gledhill JA, Moore DF, Bell D, Henry JA (1993). Subarachnoid haemorrhage associated with MDMA abuse. J. Neurol. Neurosurg. Psychiatry.

[b31] Goforth HW, Murtaugh R, Fernandez F (2010). Neurologic aspects of drug abuse. Neurol. Clin..

[b32] Hagan IG, Burney K (2007). Radiology of recreational drug abuse. Radiographics.

[b33] Halpern M, Citron BP (1971). Necrotizing angiitis associated with drug abuse. Am. J. Roentgenol. Radium Ther. Nucl. Med..

[b34] Hanyu S, Ikeguchi K, Imai H, Imai N, Yoshida M (1995). Cerebral infarction associated with 3,4-methylenedioxymethamphetamine (‘ecstasy’) abuse. Eur. Neurol..

[b35] Harries DP, De Silva RN (1992). Misuse of ecstasy. Br. Med. J.

[b36] Hart RG, Kagan-Hallet K, Joerns SE (1987). Mechanisms of intracranial hemorrhage in infective endocarditis. Stroke.

[b37] He GQ, Zhang A, Altura BT, Altura BM (1994). Cocaine-induced cerebrovasospasm and its possible mechanism of action. J. Pharmacol. Exp. Ther..

[b38] Heesch CM, Wilhelm CR, Ristich J, Adnane J, Bontempo FA, Wagner WR (2000). Cocaine activates platelets and increases the formation of circulating platelet containing microaggregates in humans. Heart.

[b39] Ho EL, Josephson SA, Lee HS, Smith WS (2009). Cerebrovascular complications of methamphetamine abuse. Neurocrit. Care.

[b40] Hughes JC, McCabe M, Evans RJ (1993). Intracranial haemorrhage associated with ingestion of ‘ecstasy’. Arch. Emerg. Med..

[b41] Ito H, Yeo KK, Wijetunga M, Seto TB, Tay K, Schatz IJ (2009). A comparison of echocardiographic findings in young adults with cardiomyopathy: with and without a history of methamphetamine abuse. Clin. Cardiol..

[b42] Jacobs IG, Roszler MH, Kelly JK, Klein MA, Kling GA (1989). Cocaine abuse: Neurovascular complications. Radiology.

[b43] Kaku DA, Lowenstein DH (1990). Emergence of recreational drug abuse as a major risk factor for stroke in young adults. Ann. Intern. Med..

[b44] Kalant H (2001). The pharmacology and toxicology of “ecstasy” (MDMA) and related drugs. Can. Med. Assoc. J..

[b45] Kaufman MJ, Levin JM, Ross MH, Lange N, Rose SL, Kukes TJ (1998). J. Am. Med. Assoc..

[b46] Klonoff DC, Andrews BT, Obana WG (1989). Stroke associated with cocaine use. Arch. Neurol..

[b47] Kolodgie FD, Farb A, Virmani R (1995). Pathobiological determinants of cocaine-associated cardiovascular syndromes. Hum. Pathol..

[b48] Kolodgie FD, Wilson PS, Cornhill JF, Herderick EE, Mergner WJ, Virmani R (1993). Increased prevalence of aortic fatty streaks in cholesterol-fed rabbits administered intravenous cocaine: the role of vascular endothelium. Toxicol. Pathol..

[b49] Kolodgie FD, Wilson PS, Mergner WJ, Virmani R (1999). Cocaine-induced increase in the permeability function of human vascular endothelial cell monolayers. Exp. Mol. Pathol..

[b50] Konzen JP, Levine SR, Garcia JH (1995). Vasospasm and thrombus formation as possible mechanisms of stroke related to alkaloidal cocaine. Stroke.

[b51] Lawson TM, Rees A (1996). Stroke and transient ischemic attacks in association with substance abuse in a young man. Postgrad. Med. J..

[b52] Lerner SE, Burns RS (1978). Phencyclidine use among youth: history, epidemiology, and acute and chronic intoxication. NIDA Res. Monogr..

[b53] Lieberman AN, Bloom W, Kishore PS, Lin JP (1974). Carotid artery occlusion following ingestion of LSD. Stroke.

[b54] Lucas CE (2005). The impact of street drugs on trauma care. J. Trauma.

[b55] Manchanda S, Connolly MJ (1993). Cerebral infarction in association with ecstasy abuse. Postgrad. Med. J..

[b56] Margolis MT, Newton TH (1971). Methamphetamine (“speed”) arteritis. Neuroradiology.

[b57] Martin-Schild S, Albright KC, Hallevi H, Barreto AD, Philip M, Misra V (2010). Intracerebral hemorrhage in cocaine users. Stroke.

[b58] Martin-Schild S, Albright KC, Misra V, Philip M, Barreto Ad, Hallevi H (2009). Intravenous tissue plasminogen activator in patients with cocaine-associated acute ischemic stroke. Stroke.

[b59] Mateo I, Pinedo A, Gomez-Beldarrain M, Basterretxea JM, Garcia-Monco JC (2005). Recurrent stroke associated with cannabis use. J. Neurol. Neurosurg. Psychiatry.

[b60] Mathew RJ, Wilson WH, Davis R (2003). Postural syncope after marijuana: a transcranial Doppler study of the hemodynamics. Pharmacol. Biochem. Behav..

[b61] McCarron MM, Schulze BW, Thompson GA, Conder MC, Goetz WA (1981). Acute phencyclidine intoxication: incidence of clinical findings in 1,000 cases. Ann. Emerg. Med..

[b62] McCarron MO, Thomas AM (1997). Cannabis and alcohol in stroke. Postgrad. Med. J..

[b63] McGee SM, McGee DN, McGee MB (2004). Spontaneous intracerebral hemorrhage related to methamphetamine abuse: autopsy findings and clinical correlation. Am. J. Forensic Med. Pathol..

[b64] Mechem CC, Hall AH (2008). CBRNE—Incapacitating agents, LSD: eMedicine emergency medicine. [homepage on the Internet]. http://emedicine.medscape.com/article/833040-overview.

[b65] Merkel PA, Koroshetz WJ, Irizarry MC, Cudkowicz ME (1995). Cocaine-associated cerebral vasculitis. Semin. Arthritis Rheum..

[b66] Mesec A, Rot U, Grad A (2001). Cerebrovascular disease associated with marijuana abuse: a case report. Cerebrovasc. Dis..

[b67] Mittleman MA, Lewis RA, Maclure M, Sherwood JB, Muller JE (2001). Triggering myocardial infarction by marijuana. Circulation.

[b68] Mokdad AH, Marks JS, Stroup DF, Gerberding Jl (2004). Actual causes of death in the United States, 2000. J. Am. Med. Assoc..

[b69] Moussouttas M (2004). Cannabis use and cerebrovascular disease. Neurologist.

[b70] Mouzak A, Agathos P, Kerezoudi E, Mantas A, Vourdeli-Yiannakoura E (2000). Transient ischemic attack in heavy cannabis smokers—how ‘safe’ is it?. Eur. Neurol..

[b71] Nolte KB, Brass LM, Fletterick CF (1995). Neurology.

[b72] Oyesiku NM, Colohan AR, Barrow DL, Reisner A (1993). Cocaine-induced aneurysmal rupture: an emergent negative factor in the natural history of intracranial aneurysms?. Neurosurgery.

[b73] Perez JA, Arsura EL, Strategos S (1999). Methamphetamine-related stroke: four cases. J. Emerg. Med..

[b74] Petitti DB, Sidney S, Quesenberry C, Bernstein A (1998). Stroke and cocaine or amphetamine use. Epidemiology.

[b75] Renard D, Taieb G, Gras-Combe G, Labauge P (2010). Cannabis-related myocardial infarction and cardioembolic stroke. J. Stroke Cerebrovasc. Dis..

[b76] Reneman L, Habraken JB, Majoie CB, Booij J, den Heeten GJ (2000). MDMA (“Ecstasy”) and its association with cerebrovascular accidents: preliminary findings. Am. J. Neuroradiol..

[b77] Rojas R, Riascos R, Vargas D, Cuellar H, Borne J (2005). Neuroimaging in drug and substance abuse part I: cocaine, cannabis and ecstasy. Top Magn. Reson. Imaging.

[b78] Salanova V, Taubner R (1984). Intracerebral haemorrhage and vasculitis secondary to amphetamine use. Postgrad. Med. J..

[b79] Shibata S, Mori K, Sekine I, Suyama H (1991). Subarachnoid and intracerebral hemorrhage associated with necrotizing angiitis due to methamphetamine abuse—an autopsy case. Neurol. Med. Chir (Tokyo).

[b80] Sloan MA, Kittner SJ, Feeser BR, Gardner J, Epstein A, Wozniak MA (1998). Illicit drug-associated ischemic stroke in the Baltimore-Washington Young Stroke Study. Neurology.

[b81] Sloan MA, Mattioni TA (1992). Concurrent myocardial and cerebral infarctions after intranasal cocaine use. Stroke.

[b82] Sobel J, Espinas OE, Friedman SA (1971). Carotid artery obstruction following LSD capsule ingestion. Arch. Intern. Med..

[b83] Stafford CR, Bogdanoff BM, Green L, Spector HB (1975). Mononeuropathy multiplex as a complication of amphetamine angiitis. Neurology.

[b84] (2010). State Medical Marijuana Laws. [homepage on the Internet]. http://www.ncsl.org/default.aspx?tabid=19587.

[b85] Stoessl AJ, Young GB, Feasby TE (1985). Intracerebral haemorrhage and angiographic beading following ingestion of catecholaminergics. Stroke.

[b86] Strote J, Lee JE, Wechsler H (2002). Increasing MDMA use among college students: results of a national survey. J. Adolesc. Health.

[b87] Substance Abuse and Mental Health Services Administration (SAMHSA) (2010). NSDUH: Latest national survey on drug use and health, substance abuse data, SAMHSA, office of applied studies. http://www.oas.samhsa.gov/nsduhLatest.htm.

[b88] (2010). The DAWN report: Highlights of the 2009 drug abuse warning network (DAWN) findings on drug-related emergency department visits.. http://dawninfo.samhsa.gov/files/SpecTopics/DAWN2010_SR034.pdf.

[b89] Togna G, Tempesta E, Togna AR, Dolci N, Cebo B, Caprino L (1985). Haemostasis.

[b90] Treadwell SD, Robinson TG (2007). Cocaine use and stroke. Postgrad. Med. J..

[b91] Volkow ND, Valentine A, Kulkarni M (1988). Radiological and neurological changes in the drug abuse patient: a study with MRI. J. Neuroradiol..

[b92] West PL, Mckeown NJ, Helman RS (2011). Phencyclidine toxicity: eMedicine Emergency Medicine. http://emedicine.medscape.com/article/816348-overview.

[b93] Westover AN, Mcbride S, Haley RW (2007). Stroke in young adults who abuse amphetamines or cocaine. Arch. Gen. Psychiatry.

[b94] Winslow BT, Voorhees KI, Pehl KA (2007). Methamphetamine abuse. Am. Fam. Physician.

[b95] Wooten MR, Khangure MS, Murphy MJ (1983). Intracerebral hemorrhage and vasculitis related to ephedrine abuse. Ann. Neurol..

[b96] Yeo KK, Wijetunga M, Ito H, Efird JT, Tay K, Seto TB (2007). The association of methamphetamine use and cardiomyopathy in young patients. Am. J. Med..

[b97] Yi SH, Ren L, Yang TT, Liu L, Wang H, Liu Q (2008). Myocardial lesions after long-term administration of methamphetamine in rats. Chin. Med. Sci. J..

[b98] Zachariah SB (1991). Stroke after heavy marijuana smoking. Stroke.

